# Nitric oxide production by *Biomphalaria glabrata *haemocytes: effects of *Schistosoma mansoni *ESPs and regulation through the extracellular signal-regulated kinase pathway

**DOI:** 10.1186/1756-3305-2-18

**Published:** 2009-04-22

**Authors:** Zahida Zahoor, Angela J Davies, Ruth S Kirk, David Rollinson, Anthony J Walker

**Affiliations:** 1 School of Life Sciences, Kingston University, Penrhyn Road, Kingston upon Thames, Surrey, KT1 2EE, UK; 2 Wolfson Wellcome Biomedical Laboratories, Department of Zoology, The Natural History Museum, Cromwell Road, London, SW7 5BD, UK

## Abstract

**Background:**

*Schistosoma mansoni *uses *Biomphalaria glabrata *as an intermediate host during its complex life cycle. In the snail, the parasite initially transforms from a miracidium into a mother sporocyst and during this process excretory-secretory products (ESPs) are released. Nitric oxide (NO) and its reactive intermediates play an important role in host defence responses against pathogens. This study therefore aimed to determine the effects of *S. mansoni *ESPs on NO production in defence cells (haemocytes) from schistosome-susceptible and schistosome-resistant *B. glabrata *strains. As *S. mansoni *ESPs have previously been shown to inhibit extracellular signal-regulated kinase (ERK) phosphorylation (activation) in haemocytes from susceptible, but not resistant, *B. glabrata *the regulation of NO output by ERK in these cells was also investigated.

**Results:**

Haemocytes from resistant snails challenged with *S. mansoni *ESPs (20 μg/ml) over 5 h displayed an increase in NO production that was 3.3 times greater than that observed for unchallenged haemocytes; lower concentrations of ESPs (0.1–10 μg/ml) did not significantly increase NO output. In contrast, haemocytes from susceptible snails showed no significant change in NO output following challenge with ESPs at any concentration used (0.1–20 μg/ml). Western blotting revealed that U0126 (1 μM or 10 μM) blocked the phosphorylation (activation) status of ERK in haemocytes from both snail strains. Inhibition of ERK signalling by U0126 attenuated considerably intracellular NO production in haemocytes from both susceptible and resistant *B. glabrata *strains, identifying ERK as a key regulator of NO output in these cells.

**Conclusion:**

*S. mansoni *ESPs differentially influence intracellular NO levels in susceptible and resistant *B. glabrata *haemocytes, possibly through modulation of the ERK signalling pathway. Such effects might facilitate survival of *S. mansoni *in its intermediate host.

## Background

Nitric oxide (NO) is a highly reactive molecule produced by mammalian, invertebrate and plant cells. In mammals, NO functions as a neuronal messenger molecule, a vasodilator of smooth muscle, a regulator of cell proliferation and apoptosis, and a cytotoxic effector [1]. NO is synthesised by the oxidation of L-arginine to L-citrulline, catalysed by the enzyme NO synthase (NOS). In mammals, three isoforms of NOS have been characterised; neuronal NOS (nNOS) and endothelial NOS (eNOS), which are constitutively expressed, and inducible NOS (iNOS), which is expressed in response to inflammation and proinflammatory cytokines [2]. NO and its reactive intermediates can damage enzymes and DNA and are thus produced by host cells to protect the host against a range of pathogens [3]. NOS is a conserved enzyme with a great degree of sequence similarity between invertebrates and vertebrates [4]. In molluscs, NOS-like activity has been identified in *Mytilus galloprovincialis*, *Lymnaea stagnalis *and *Biomphalaria glabrata *defence cells (haemocytes); while nNOS has been found in *L. stagnalis *and *Aplysia californica *neurons [5-11]. To date, limited information is available on how molluscan haemocytes regulate NO production under normal and stressed conditions.

A number of complex interactions exist between *Schistosoma mansoni *and its molluscan intermediate host *B. glabrata*, from initial infection with miracidia to the development of mother sporocysts, subsequent daughter sporocysts, and finally the production and release of cercariae from the snail [12]. *Schistosoma mansoni *excretory-secretory products (ESPs) generated during the miracidium to mother sporocyst transition are known to modulate a range of physiological functions in *B. glabrata *haemocytes [13,14]. To date, the effect of *S. mansoni *ESPs on haemocyte NO generation in snails has not been investigated. In the present study it is shown that haemocytes from schistosome-susceptible and schistosome-resistant strains of *B. glabrata *generate different levels of basal NO and that *S. mansoni *ESPs increase intracellular NO production in haemocytes from resistant snails only. Previous studies have shown that the extracellular signal-regulated kinase (ERK) signalling pathway plays an important role in controlling NO and hydrogen peroxide (H_2_O_2_) output in *L. stagnalis *haemocytes [5,15]. Furthermore, *S. mansoni *ESPs (10 – 20 μg/ml) have been found to inhibit ERK signalling in haemocytes from schistosome-susceptible, but not resistant, *B. glabrata *[16]. In the present study, we sought to investigate the effect of ERK inhibition on NO output in these *B. glabrata *strains.

## Results

**Figure 1:**
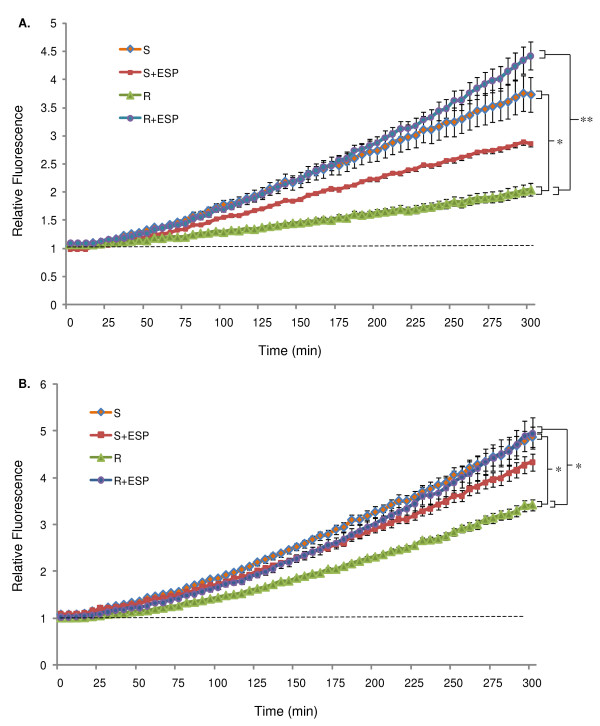
**Relative NO produced in haemocytes from schistosome-susceptible (S) and schistosome-resistant (R) *B. glabrata *strains in the presence and absence of 20 μg/ml ESPs over time**. Equal volumes (100 μl per well) of diluted haemolymph **(A)**, or an equal number of haemocytes (3 × 10^3 ^cells per well) **(B) **from each snail strain were used in NO assays. Mean relative fluorescence from a single pool of haemolymph (± SEM, n = 4) is shown for each time point as a proportion of background fluorescence (no DAF-FM diacetate, indicated by the dotted line). Data shown are from one assay and are representative of three different assays conducted on separate days. *P ≤ 0.05 and **P ≤ 0.01 for differences in mean fluorescence values between treatments at 2 h.

The relative amount of intracellular NO produced by haemocytes from susceptible and resistant snail strains in the presence of 20 μg/ml ESPs compared to saline-only controls was investigated over 5 h, using equal amounts of haemolymph per well. This was to compare responses in a physiological context, without accounting for the fact that resistant snail strains generally have twice as many haemocytes per volume of haemolymph compared with susceptible snails of similar age and size [17]. Figure 1A shows that ESPs significantly increased NO production in haemocytes from resistant snails only (*P *≤ 0.01). After 5 h of ESP challenge, the increase in mean NO production (from 0 min) by haemocytes from resistant snails was 3.3 times that of saline-only controls (mean relative increases of 3.420 and 1.045, respectively). In contrast, mean NO output by haemocytes from susceptible snails decreased slightly when challenged with ESPs; although this difference was not statistically significant, a decrease was observed consistently from three biological replicates. *Schistosoma mansoni *ESPs (20 μg/ml) gave little or no auto-fluorescence over time, thus the presence of ESPs in the assay did not affect the fluorescence values. Interestingly, there was a considerable difference in the basal NO outputs produced over time between unchallenged haemocytes from resistant and susceptible snail strains, with haemocytes from susceptible snails producing 2.6 times more NO than haemocytes from resistant snails after 5 h of incubation (mean relative increases of 2.73 and 1.04, respectively) (Fig. 1A). Viability assays were used to assess haemocyte survival after 5 h incubation in medium only (CBSS) or CBSS with 20 μg/ml ESPs. Both trypan blue alive/dead cell estimations and the CellTiter 96 AQueous One Solution Assay showed haemocytes remained 100% viable after 5 h *in vitro *incubation in the presence and absence of 20 μg/ml ESPs.

In order to compensate for the fact that resistant snails generally have twice as many haemocytes compared with susceptible snails [17], an equal number of haemocytes was used from each snail strain in all subsequent experiments. Haemocytes from resistant snails exposed to ESPs produced 0.6 times more NO over 5 h than unexposed controls (mean relative increases of 3.96 and 2.42, respectively) (Fig. 1B). Nitric oxide production by haemocytes from susceptible snails was not affected significantly by ESP challenge. Even when accounting for haemocyte numbers, basal NO levels in susceptible snail haemocytes were higher than in resistant snail haemocytes after 5 h incubation in saline.

**Figure 2:**
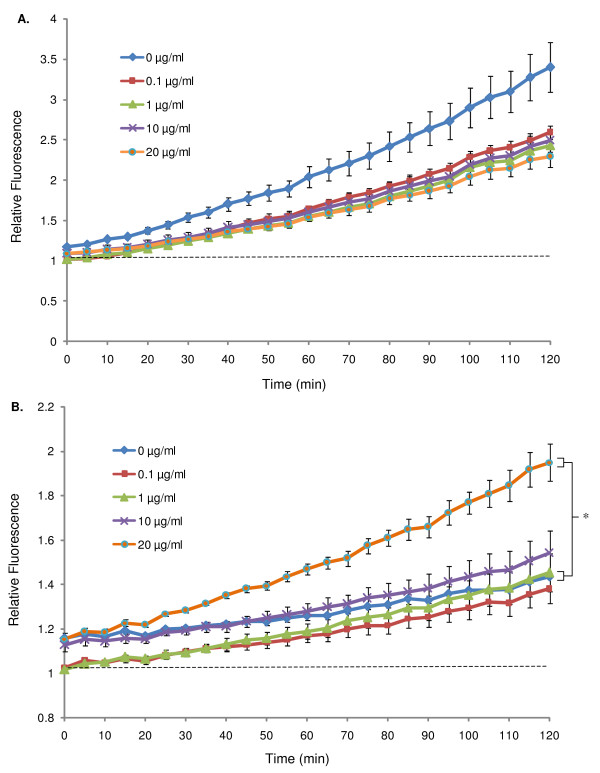
**Relative NO produced in haemocytes from schistosome-susceptible (A) and schistosome-resistant (B) *B. glabrata *strains challenged with different concentrations of ESPs (0, 0.1, 1, 10 and 20 μg/ml) over time**. Equal numbers of haemocytes (3 × 10^3 ^cells per well) from each snail strain were used in each experiment. Mean relative fluorescence from a single pool of haemolymph (± SEM, n = 3) is shown for each time point as a proportion of background fluorescence (no DAF-FM diacetate, indicated by the dotted line). Data shown are from one assay and are representative of three different assays conducted on separate days. *P ≤ 0.05 for differences in mean fluorescence values at 2 h between unexposed haemocytes and haemocytes exposed to ESPs.

To determine the dose dependency of the NO response to ESPs, haemocytes extracted from either susceptible or resistant snails were challenged with different ESP concentrations (0.1–20 μg/ml). As a general linear relationship between NO generation by haemocytes and time was observed during the first 2 h incubation in previous experiments (Fig. 1), relative NO levels were subsequently measured every 5 min for this duration. ESP concentrations of 0.1–20 μg/ml did not significantly affect mean NO output in haemocytes from susceptible snails compared to unexposed controls (Fig. 2A). A clear dose response was not observed when haemocytes from resistant snails were exposed to different ESP concentrations; only 20 μg/ml ESPs significantly enhanced mean relative NO output (*P *≤ 0.05) (Fig. 2B).

**Figure 3:**
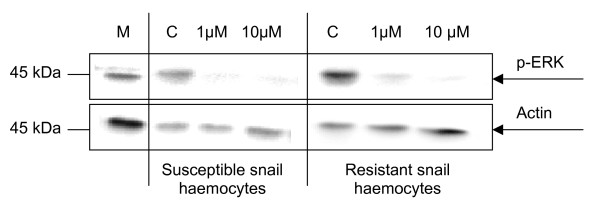
**Western blot showing phosphorylated ERK (p-ERK) levels in haemocytes from schistosome-susceptible and schistosome-resistant *B. glabrata *strains**. Haemocytes were exposed to the MEK1/2 inhibitor, U0126 (1 μM or 10 μM) for 20 min before cell lysis. Unexposed haemocytes (C) and a mammalian (M) cell line (HC60) were used as controls. Results are representative of two independent experiments.

**Figure 4:**
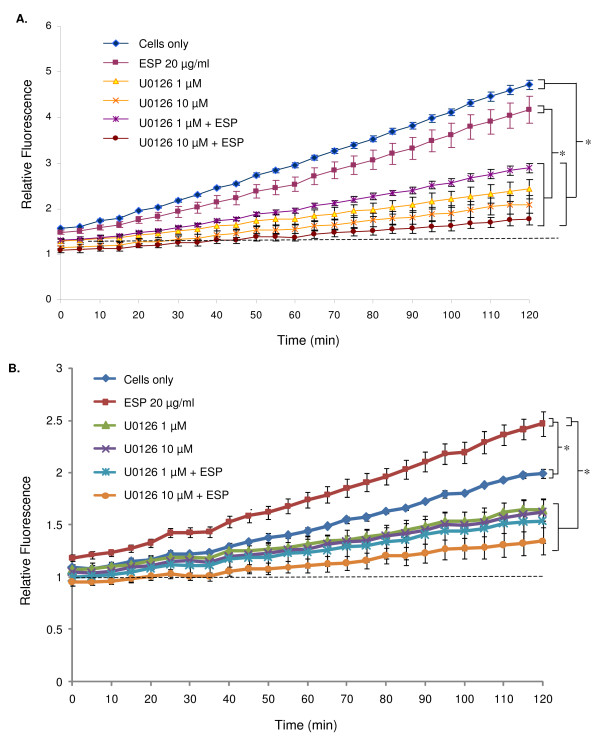
**Relative NO levels in haemocytes from (A) schistosome-susceptible and (B) schistosome-resistant *B. glabrata *following exposure to ESPs (20 μg/ml), U0126 (1 μM or 10 μÌ) or both**. Equal numbers of haemocytes (3 × 10^3 ^cells per well) from susceptible or resistant snails were exposed to U0126 (1 μM or 10 μM), ESPs (20 μg/ml), or both. Mean relative fluorescence from a single pool of haemolymph (± SEM, n = 3) is shown for each time point as a proportion of background fluorescence (no DAF-FM diacetate indicated by a dotted line). Data shown are from one assay and are representative of three different assays conducted on separate days. *P ≤ 0.05 for differences in mean fluorescence values between treatments at 2 h.

In order to investigate whether the ERK cell signalling pathway plays a role in regulating NO production in *B. glabrata *haemocytes, cells were pre-incubated with U0126, a highly selective inhibitor of mitogen-activated protein kinase kinase (MEK1/2), the upstream kinase responsible for ERK phosphorylation (activation). This inhibitor has been used previously in studies with *L. stagnalis *haemocytes [18,5]. Western blot analysis (Fig. 3) and densitometric analysis (data not shown) highlighted that 1 μM or 10 μM U0126 inhibited the phosphorylation of ERK in both snail strains by 80–85%. Inhibition of ERK by U0126 coincided with a significant decrease in NO production in both snail strains over 2 h (*P *≤ 0.05) (Fig. 4). Haemocytes from susceptible snails showed a 64% decrease in NO output over 2 h in the presence of 1 μM U0126 compared to controls (Fig. 4A). The presence of both U0126 and 20 μg/ml ESP did not further reduce NO levels compared to U0126 (1 μM or 10 μM) alone (Fig. 4A). Haemocytes from resistant snails challenged with U0126 exhibited a 60% decrease in NO levels over 2 h compared to controls without inhibitor. Haemocytes exposed to 10 μM U0126 and 20 μg/ml ESP displayed the greatest reduction in NO output, with mean values almost reduced to background levels (Fig. 4B).

## Discussion

The effect of ESPs on haemocyte NO production is strain specific; haemocytes from schistosome-resistant snails displayed significantly increased NO output after ESP challenge. Previous studies using the same snail strains have highlighted *S. mansoni *ESPs' ability to attenuate ERK signalling in haemocytes from the susceptible *B. glabrata *strain, with no effect in those from the resistant strain [16]. In the present study, inhibition of ERK signalling in haemocytes from both snail strains led to down-regulation of NO production, demonstrating that NO production is partly under the control of ERK signalling, which is consistent with earlier work on *L. stagnalis *haemocytes [5].

A highly sensitive NO probe (DAF-FM diacetate) which detects intracellular NO was used in this investigation. This probe has been used previously in other invertebrate models including: the squid, *Euprymna scolopes*, the moth, *Manduca sexta*, the sea squirt,* Ciona intestinalis *and the snail *L. stagnalis *[5,19-21]. In molluscs, the production of NO has also been studied in the nervous system [10,11]. Franchini *et al*., [22] identified an immunoreactive NOS-like protein in molluscan haemocytes that could be induced by stimulating the cells with *E. coli*, while haemocytes from the bivalve *M. galloprovincialis *were shown to generate superoxide and nitrites following exposure to phorbol myristate acetate (PMA), laminarin and during yeast cell phagocytosis [23]. Lipopolysaccharide (LPS) does not induce NO generation in *L. stagnalis *or *M. galloprovincialis *haemocytes [5,6], but does stimulate an NO response in the clam *Ruditapes decussates *[24].

Oxygen-dependent killing mechanisms in molluscs such as *B. glabrata *are known to play a crucial role in killing schistosome sporocysts [7,25,26]. It has been hypothesised previously that differences exist between schistosome-resistant and schistosome-susceptible *B. glabrata *strains in their oxygen-dependent killing mechanisms [27]. *Schistosoma mansoni *ESPs are known to affect the physiology of haemocytes; cell motility, phagocytosis of zymosan particles, and the production of reactive oxygen metabolites are differentially modulated in susceptible and resistant *B. glabrata *strains by *S. mansoni *ESPs [28-30].

In the present study, NO levels in *B. glabrata *haemocytes from susceptible and resistant snail strains were found to be affected differentially by *S. mansoni *ESPs. Haemocytes from resistant snails had a significantly greater increase (3.3 times) in NO production than controls following 5 h ESP challenge, while haemocytes from susceptible snails were not significantly affected. Furthermore, basal levels of NO (from unchallenged haemocytes) were significantly different between the two snail strains, with haemocytes from susceptible snails producing relatively more NO over time. The reason for the basal NOS activity in extracted haemocytes is unknown; similar basal activities were also observed previously in extracted *L. stagnalis *haemocytes [5]. Earlier studies have shown that haemocytes from schistosome-resistant *B. glabrata *strains maintain higher levels of intracellular superoxide and hydrogen peroxide when stimulated with *S. mansoni *ESPs and PMA, respectively, compared to susceptible strains [28,31]. Hahn *et al*. [26] reported no differences in the relative production of reactive oxygen species (ROS) in haemocytes from susceptible and resistant snail strains following stimulation with carbohydrates known to be present on the schistosome surface, while Humphries and Yoshino [32] reported no effect of *S. mansoni *ESPs on hydrogen peroxide generation in haemocytes from resistant *B. glabrata*.

Human recombinant interleukin-2 (IL-2), a known NO stimulant for mammalian macrophages, was found to enhance considerably NO production in *M. galloprovincialis *haemocytes by approximately 13-fold, an effect which was reduced in the presence of a protein kinase A (PKA) inhibitor [6]. Thus a cAMP-dependent protein kinase might be involved in NO generation in molluscs, together with PKC, which was found to be an important NO regulator in *L. stagnalis *haemocytes [5]. Here, in the presence or absence of ESPs, the MEK inhibitor, U0126, significantly reduced NO production in susceptible and resistant *B. glabrata *haemocytes. The inhibitor also substantially attenuated ERK phosphorylation in haemocytes from both snail strains. This implies a role for ERK signalling in NO output through NOS regulation, similar to that reported in *L. stagnalis *haemocytes [5]. Extracellular hydrogen peroxide generated by stimulating *B. glabrata *haemocytes with PMA, galactose-conjugated BSA, or through the process of encapsulation and phagocytosis is also partially regulated by ERK signalling [32,33].

Earlier work has shown that haemocytes from schistosome-susceptible *B. glabrata *challenged with ESPs display significantly reduced ERK phosphorylation [16]. In the current investigation, haemocytes from the same snail strain had significantly reduced NO levels following exposure to the ERK inhibitor, U0126. However, reduced NO output in the presence of U0126 could also be attributed to an effect of U0126 on an ERK-like protein, not recognised by the anti-phospho ERK antibody used here and in the study by Zahoor *et al*. [16]; only one ERK-like protein was detected by this antibody in *B. glabrata *haemocytes extracts, whereas two ERK isoforms are sometimes detected in *L. stagnalis *[18]. Moreover, the increased NO output observed here in resistant snail haemocytes following ESP exposure might be a consequence of the sustained ERK phosphorylation previously seen in these cells under ESP challenge [16]. In addition, ESPs may be influencing the activities of other cell signalling pathways, such as protein kinase C (PKC) or PKA, resulting in modulation of intracellular NO production.

Given the cytotoxic effects of NO, longer-term effects of ESPs on NO production in haemocytes from both snail strains may influence survival of invading schistosomes *in vivo*. In the present *in vitro *study, schistosome-susceptible snail haemocytes have been shown to produce more intracellular NO under basal conditions than those of resistant snails (even though resistant snails have been reported to possess twice as many haemocytes [17]). Why this phenomenon exists is currently unknown, but may involve the haemocytes maintaining different intracellular NO equilibriums. Intracellular ROS equilibriums are known to play an important role in allowing long term survival of the host and the parasite; in the case of malaria, increased oxidative stress in the host's erythrocytes can lead to exacerbated disease progression, while partial inhibition of macrophage NO production by *Toxoplasma gondii *enhances the parasite's survival [34,35]. Furthermore, an intracellular NO equilibrium may be physiologically important; NO is involved in a number of cell signalling events and, depending on the cellular environment, can promote either cell survival or cell death [36,37].

## Conclusion

Investigation of NO generation by haemocytes from schistosome-susceptible and schistosome-resistant *B. glabrata *has revealed notable strain-dependent differences in the capacities of these cells to produce NO under basal conditions, and in the presence of *S. mansoni *ESPs. Haemocyte NO output appears to be regulated by the ERK signalling pathway, which might be important for the outcome of infection, particularly as *S. mansoni *differentially influences ERK activation in these defence cells [16]. Although it is acknowledged that invading schistosomes employ multiple strategies to evade snail-host defence mechanisms, our understanding of such interactions remains rudimentary. Only through further integrative and functional investigations of the many interactions that occur between haemocytes and schistosomes will we begin to unravel the real breadth of the interactions occurring between the developing schistosome and its snail host.

## Methods

### Animals

Snails used in this study included a *B. glabrata *strain resistant to *S. mansoni *(NHM3017), originally derived from BS90, and a *B. glabrata *strain susceptible to *S. mansoni *(NHM1742). All snails were maintained at 26°C with a 12 h: 12 h, light: dark cycle, and were fed fresh round lettuce twice-weekly. The life cycle of *S. mansoni *(Belo Horizonte strain) was maintained in albino CD1 mice; animal use received appropriate ethical approval.

### Collection of *S. mansoni *ESPs

*In vitro *transformation of *S. mansoni *miracidia into mother sporocysts and collection of *S. mansoni *ESPs have been described previously [16]. Briefly, eggs from five *S. mansoni-*infected mice livers and spleens were isolated and placed in spring water (Evian). Hatched miracidia were then collected using a pipette and were washed in spring water and concentrated using a Stericup HV filter unit, with a 0.45 μm membrane (Millipore, Watford, United Kingdom). Approximately 60,000 miracidia were left to transform in 25 cm^2 ^vented sterile tissue culture flasks (Nunc, Rochester, USA) at 26°C for 36–40 h in sterile Chernin's balanced salt solution (CBSS) [38] with glucose and trehalose (1 g/L each) and penicillin and streptomycin (100 U/ml of each). All chemicals were purchased from Sigma, Poole, United Kingdom. Once the miracidia had transformed into mother sporocysts, each culture flask was shaken gently to remove any remaining miracidial ciliated plates from the sporocysts' surface. After the sporocysts and ciliated plates had settled on the base of the flask, the culture medium containing the ESPs was collected and centrifuged briefly at 10,000 × *g *to remove any particulates. The supernatant was then concentrated approximately 10–20 times at 4°C, in Vivapore 10 ml concentrators (Vivascience, Sartorius, Epsom, UK) with a 7,500 molecular weight cut off. The protein concentration of the ESP preparation was then determined with the NanoOrange fluorescence-based protein assay kit (Molecular Probes, Leiden, Netherlands) and a Fluorstar Optima microplate spectrofluorometer (BMG Labtech, Aylesbury, UK), using bovine serum albumin (BSA) as the protein standard. The ESP solution was aliquoted and stored at -20°C.

### Nitric oxide assay

Haemolymph was extracted from *B. glabrata *using the head-foot retraction method, pooled, and diluted in CBSS (2 parts haemolymph: 1 part CBSS). Either 100 μl of diluted haemolymph (containing approximately 7.5 × 10^3 ^haemocytes in resistant samples or 4 × 10^3 ^cells in susceptible samples) or approximately 3 × 10^3 ^cells per well from each snail strain were used to create cell monolayers in the individual wells of 96 well culture plates (Corning Costar, Schiphol-Rijk, The Netherlands). Estimation of cell numbers was performed using disposable haemocytometers (Immune systems Ltd., Paignton, UK). Haemocytes were left to adhere for 30 min at room temperature (RT), subsequently the haemolymph was removed and the cell monolayer washed twice with 250 μl CBSS. A fluorescent dye, 4-Amino-5-methylamino-2',7'-difluorofluorescein (DAF-FM) diacetate (Molecular Probes), which is highly sensitive to intracellular NO, was dissolved in dimethyl sulfoxide (DMSO) and added to the cell monolayer at 5 μM (final DMSO concentration 0.1% (v/v)) for 1 h in the dark at RT. Cells were then washed twice with 100 μl CBSS before being challenged with *S. mansoni *ESPs (0–20 μg/ml). These ESP concentrations were chosen as they are similar to those used in a previous study in which ESPs were shown to differentially module ERK signalling in haemocytes from the same snail strains [16]. The controls included haemocytes only to account for background fluorescence, and ESPs only (20 μg/ml). The fluorescent signal was then measured in a Fluorstar Optima microplate reader at 485 nm excitation and 520 nm emission every 5 min. Each assay was conducted on one day in triplicate or quadruplicate using samples from the same pool of haemolymph. Assays were then repeated on different days using different pooled haemolymph samples to ensure consistency. Because background fluorescence varied in each assay, data from separate assays could not be combined; thus for each experiment one typical graph (from three) is shown together with the statistical analysis.

The ERK pathway inhibitor, U0126 (New England BioLabs, Hitchin, UK), was dissolved in DMSO and used at a final concentration of 1 μM or 10 μM in CBSS (final DMSO concentration 0.1% (v/v)). This inhibitor has been used previously to block MEK1/2 phosphorylation (activation) in molluscan haemocytes [5,16,18]. Cell monolayers were incubated with U0126 for 20 min after removal of DAF-FM diacetate (as described above), prior to ESP (20 μg/ml) challenge.

### Cell viability assay

Extracted haemocytes (in the presence and absence of 20 μg/ml ESPs) were assessed for their viability after 3–5 h *in vitro *culture in 96 well plates. Percentage cell death was calculated based on the uptake of 0.2% trypan blue. A commercially available CellTiter 96 AQueous One Solution Cell Proliferation Assay (Promega, Southampton, UK) was also used to monitor cell viability; the assay contains a tetrazolium compound that is bio-reduced by active cells into formazan. Cell monolayers were incubated in either 100 μl CBSS, or 100 μl ESP (20 μg/ml) in CBSS; 20 μl of CellTiter 96 AQueous One Solution reagent was pipetted into each well and left for 1 h before the absorbance (at 490 nm) was measured over 5 h.

### Detection of phosphorylated ERK by western blotting

Diluted haemolymph (250 μl) (2 parts haemolymph: 1 part CBSS) was pipetted into individual wells of a 48 well flat bottom culture plate (Corning Costar) and haemocytes were left to adhere for 30 min at RT. The haemolymph was then removed and the cell monolayer washed twice with CBSS before U0126 was added to the wells at 1 μM, or 10 μM, for 20 min at RT. The medium was removed and 25 μl of boiling 1 × SDS-PAGE sample buffer was added to the monolayers to solubilize haemocyte proteins. Samples were sonicated for 30 s and boiled for 5 min; once cooled, the samples were loaded onto discontinuous SDS-PAGE gels, containing 10% acrylamide in the resolving gel. A protein extract from a mammalian cell line (HC60) was also loaded onto the gel as a positive control. Separated proteins were then electrophoretically transferred onto a Hybond nitrocellulose membrane (0.45 μm; Amersham Bioscience, Amersham, UK), which was blocked for 1 h at RT with 5% (w/v) non-fat dried milk in Tris-buffered saline (TBS) containing 0.1% (v/v) Tween-20 (TTBS). The membrane was incubated overnight at 4°C in anti-phospho-p44/42 (Thr202/Tyr204) MAPK primary antibody (New England BioLabs, 1/1000 in TTBS containing 1% (w/v) BSA) and was subsequently washed three times in TTBS. This antibody has been used previously to detect molluscan phosphorylated (activated) ERK-like proteins [5,16,18,39]. Next, the membrane was incubated with an anti-rabbit horseradish peroxidase (HRP)-conjugated secondary antibody (1/2500 in TTBS; Sigma) for 2 h at RT. The signal was developed using SuperSignal West Pico chemiluminescent substrate (PerBioscience, Tatenhall, UK) and captured using a GeneGnome Bio Imaging System (Syngene, Cambridge, UK) and Syngene software. Blots were stripped of antibodies using Restore western blot stripping buffer (PerBioscience) for 3 h at RT, washed with TTBS, and incubated in anti-actin rabbit polyclonal antibodies (1/2500 in TTBS; Sigma) overnight at 4°C, prior to detection with anti-rabbit HRP-conjugated secondary antibodies (1/2500 in TTBS; Sigma) and chemiluminescent substrate; this step was to confirm equal loading of protein between lanes.

### Statistical analysis

Data from each NO assay was imported into Microsoft Excel for analysis. Relative fluorescence was calculated by dividing the fluorescence values obtained at each time point with background fluorescence levels (cells only with no DAF-FM diacetate). Where appropriate, paired Student's t-test was used to calculate statistical significance at 2 h between samples.

## Competing interests

The authors declare that they have no competing interests.

## Authors' contributions

ZZ carried out the experimental work, interpreted and analysed the data and wrote the manuscript. AJW conceived the project, provided technical support and helped draft the manuscript. AJD conceived the project and provided significant support to the preparation of the manuscript. RSK conceived the project and critically revised the manuscript. DR conceived the project, critically revised the manuscript and gave the final approval of the version to be published. All authors read and approved the final manuscript.
